# A general strategy for synthesis of metal oxide nanoparticles attached on carbon nanomaterials

**DOI:** 10.1186/1556-276X-6-71

**Published:** 2011-01-12

**Authors:** Yi Zhao, Jiaxin Li, Chuxin Wu, Lunhui Guan

**Affiliations:** 1 State Key Lab of Structural Chemistry, Fujian Institute of Research on the Structure of Matter, Chinese Academy of Sciences, YangQiao West Road 155#, Fuzhou, Fujian 350002, P. R. China

## Abstract

We report a general strategy for synthesis of a large variety of metal oxide nanoparticles on different carbon nanomaterials (CNMs), including single-walled carbon nanotubes, multi-walled carbon nanotubes, and a few-layer graphene. The approach was based on the π-π interaction between CNMs and modified aromatic organic ligands, which acted as bridges connecting metal ions and CNMs. Our methods can be applicable for a large variety of metal ions, thus offering a great potential application.

## Introduction

The attachment of nanoparticles (NPs) on carbon nanomaterials (CNMs), including single-walled carbon nanotubes (SWNTs), multi-walled carbon nanotubes (MWNTs), and graphene has attracted great interest, for the nanocomposites not only combine the extraordinary properties of the NPs and CNMs, but also exhibit some new properties caused by the interaction between them [1,2]. For examples, when the light-harvesting NPs, such as TiO_2_, ZnO, CdS, CdSe, were attached on carbon nanotubes (CNTs) with high conductivity, the photocatalytic properties increased dramatically [3-5]. In addition, CNTs with large surface areas are ideal supporting materials for catalysts NPs, leading to improvements in the efficiency of the catalysts [6-8]. A lot of approaches including assembling pre-synthesized NPs as building blocks on CNTs, and spontaneous formation of NPs on CNTs, have been applied to prepare NPs/CNTs [9-14]. The previous reports mainly focused on attaching NPs on MWNTs by using benzyl alcohol or pyrene derivatives as linkages [15,16]. Development to SWNTs and graphene, both with well-defined structures, may provide important information to explore the mechanisms of the enhanced properties of NPs after attached on CNMs. However, it still remains a challenge to fabricate uniform NPs/CNMs nanocomposites in a controlled manner. Here we present a unified strategy for synthesis of a large variety of NPs of metal oxides, including transition and rare earth metal oxides on SWNTs, MWNTs, and a few-layer graphene. The strategy was based on a noncovalent π-π interaction between delocalized π-electrons of CNMs and aromatic organic compounds, in this case phenylphosphonic acid, which acid tail can be connected with metal ions. After a hydrothermal treatment, the metal oxides NPs were formed and strongly anchored to the surface of CNMs.

## Experimental sections

**Figure 1:**
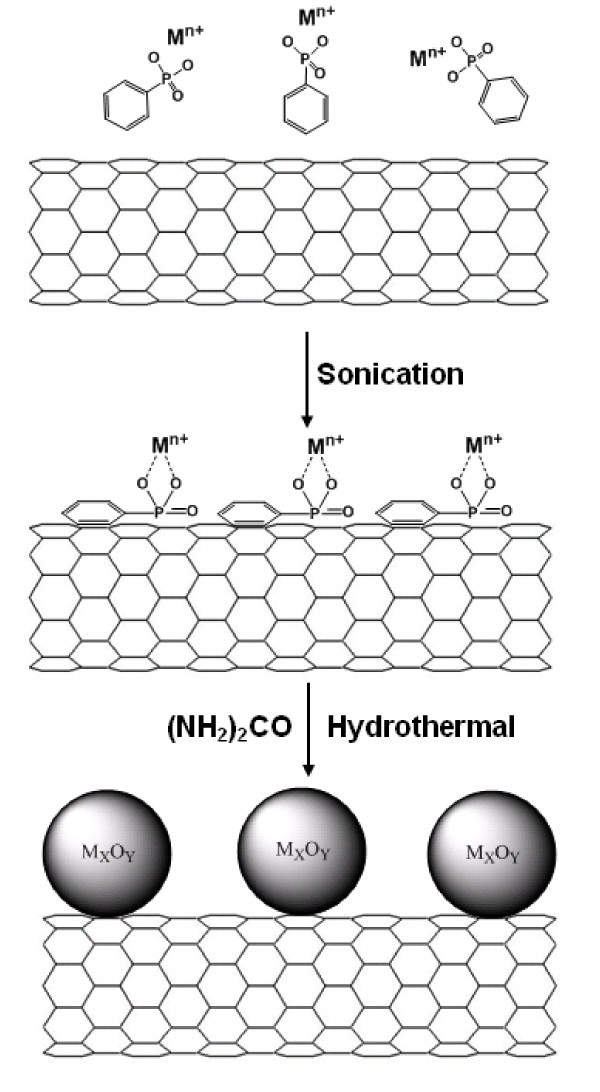
**A schematic representation of attaching various metal oxide NPs on different CNMs**.

In our experiments, MWNTs (purity 95%, 20-30 nm in diameters) were obtained from Shenzhen Nanotech Port (Shenzhen, China) and used as received, SWNTs (purity 99%, 1.4 nm in diameter) were produced by our recent methods [17], and graphene was produced by a modified arc-discharged [18]. The experimental scheme is shown in Figure 1: metal ions were ligated by phenylphosphonic acid, which was then connected with CNMs via noncovalent π-π interaction after sonication, then (NH_2_)_2_CO was added. The solution was transferred to an autoclave and incubated by a hydrothermal treatment. The hydrothermal reaction of metal ions and urea will result in the formation of metal oxide NPs [19]. The final precipitates were filtered and washed several times with water. The samples were characterized by transmission electron microscope (TEM), X-ray diffraction (XRD), and thermogravimetric analysis (TGA). See Additional file 1 (SI 1) for more experimental details. In this study, phenylphosphonic acid played a key role on attaching NPs on CNMs. For comparison, a TEM image of the typical products without phenylphosphonic acid was shown in Additional file 1 (SI 2). The particles size was obviously larger and did not connect with SWNTs. We also checked the intermediate product after sonication by TGA. The TGA measured the total metal content with a heating rate of 10°C/min in air. The results proved that there was weak interaction between metal ions and CNMs without phenylphosphonic acid. The TGA residue (mainly iron oxide) of the products, made from SWNTs sonicated with only Fe^3+^, was nearly zero. The results proved that without phenylphosphonic acid, the interaction between Fe^3+ ^and SWNTs was so weak that the meal ions were easily washed away. On the contrary, the resulting residue from SWNTs sonicated with Fe^3+ ^was around 20%. The results provided direct evidence that phenylphosphonic acid acted as bridges connecting metal ions and CNMs.

## Results and discussion

**Figure 2:**
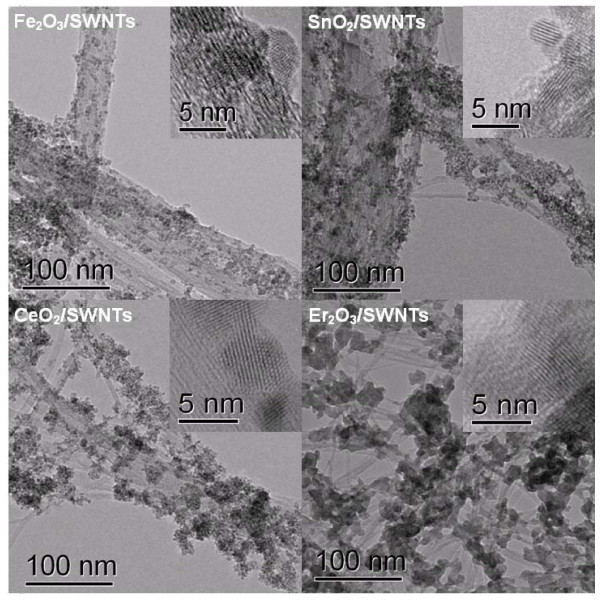
**TEM images of various metal oxide NPs of Fe_2_O_3_, SnO_2_, CeO_2_, and Er_2_O_3 _on SWNTs**.

Figure 2 shows TEM images of typical samples of Fe_2_O_3_, SnO_2_, CeO_2_, and Er_2_O_3 _on SWNTs, respectively. The SWNTs without NPs attachment were seldom observed by TEM observation. The sizes and loading ratio of NPs on SWNTs can be controlled by altering temperature, the ligand, and the initial concentrations of the metal ions. It is worth to note that the loading ratio in Figure 2 was relatively high, around 80%, resulting in the agglomerating of the NPs on the CNMs. The interface between NPs and CNMs is not prominent. When we decreased the loading ratio, the uniformly dispersed NPs were appeared on the surface of CNMs. See Additional file 1 (SI 3) for the SnO_2 _on SWNTs as example. Inserted images corresponding to their high resolution (HR) TEM images indicated that the metal oxide NPs were usually in round shapes binding on SWNTs. HR-TEM images revealed the detailed structures of these nanocrystals. Typical HR-TEM image of Fe_2_O_3 _nanocrystals with diameters of approximately 4 nm presents a crystal lattice of approximately 0.25 nm, corresponding to (110) planes of α-Fe_2_O_3_. The result was accorded with XRD pattern shown in Additional file 1 (SI 3). The regular interplanar spacing of 0.33 nm for SnO_2_, 0.27 nm for CeO_2_, was ascribed to (110) planes of SnO_2_, (200) planes of CeO_2_, respectively. However, as for the rare earth metal oxide Er_2_O_3_, it did not form fine crystalline structures in such reaction conditions. The result was confirmed by the powder XRD pattern shown in Additional file 1 (SI 3). One might expect formation of crystalline Er_2_O_3 _NPs after thermal annealing. The nanohybrid materials have many potential applications compared with the isolated NPs, because SWNTs act as carrier to stabilize NPs, maintaining their integrity. We selected Fe_2_O_3_/SWNTs as a model case for superior anode materials of lithium ion batteries.

**Figure 3:**
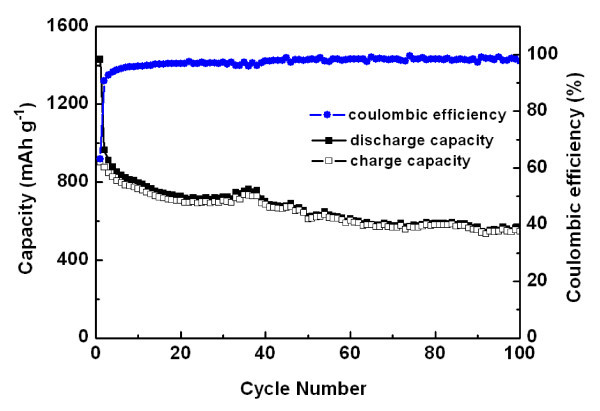
**Cycle performance and columbic efficiency of Fe_2_O_3_/SWNTs nanohybrids with a current density of 150 mA g^-1^**.

Figure 3 displays the high reversibility of the electrochemical reactions of Fe_2_O_3_/SWNTs nanohybrid over many charge-discharge cycles and the columbic efficiency. After 100 cycles at 150 mA g^-1^, it still remained a high reversible capacity of 560 mAh g^-1^, which was significantly higher than that of graphite (372 mAh g^-1^) and Fe_2_O_3 _nanotube (510 mAh g^-1 ^at 100 mA g^-1^) [20]. The columbic efficiency of the whole 100 cycles was around 97%. Our previous results indicated that the SWNTs produced by our method provided low Li insertation/de-insertation capabilities, around 200 mAh g^-1 ^[21], so the superior capabilities of Fe_2_O_3 _NP/SWNTs electrode were ascribed to the reactions involving Fe2^+^-Fe3^+ ^conversions. The performance of the nanocomposites was mainly determined by the particle sizes and loading ratio of the NPs.

**Figure 4:**
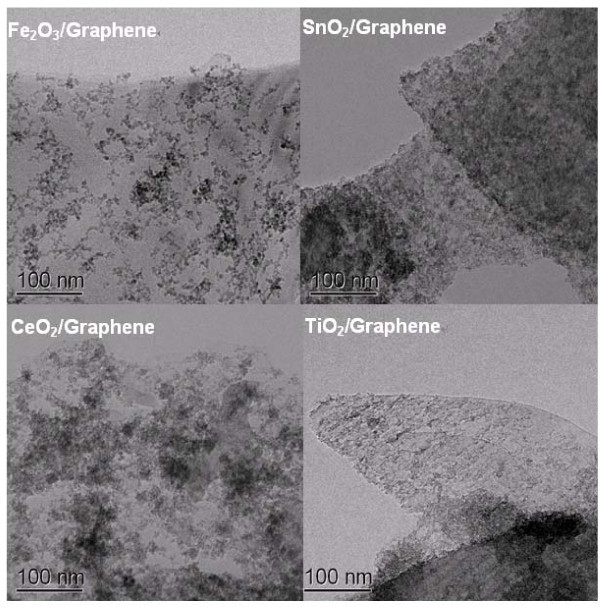
**TEM images of various metal oxide NPs of Fe_2_O_3_, SnO_2_, CeO_2_, and TiO_2 _on a few-layer graphene**.

Our method was based on π-π interaction between ligand and CNMs, thus can also be generally applicable to graphene and MWNTs. Shown in Figure 4 are TEM images of Fe_2_O_3_, SnO_2_, CeO_2_, and TiO_2 _NPs assembled with a few-layer graphene. The diameters and loading ratio of NPs were controlled by temperature and the mole ratio of metal ions to graphene nanosheets. Typically, the particles are remarkably smaller when deposited on CNMs compared with unanchored phase, since CNMs can prevent crystal growth during crystallization. We also succeeded in introducing NPs of different rare earth metal oxides on MWNTs. See Additional file 1 (SI 5) for more details.

## Conclusion

In summary, we report a general strategy for synthesis of a large variety of metal oxide NPs on CNMs, including SWNTs, MWNTs, and a few-layer graphene. The approach was based on the π-π interaction between CNMs and modified aromatic organic ligands, which acted as bridges connecting metal ions and CNMs. Our methods can be applicable for a large variety of metal ions. By adopting bi-metal or even tri-metal precursors in a certain mole ratio, composite oxide nanocrystals with novel structures and multi-function deposited on different CNMs can be effectively prepared through this method. The new class of hybrid nanomaterials offers a great potential application in sustainable energy, environment, and even biomedicine.

## Abbreviations

CNMs: carbon nanomaterials; CNTs: carbon nanotubes; MWNTs: multi-walled carbon nanotubes; NPs: nanoparticles; SWNTs: single-walled carbon nanotubes; TEM: transmission electron microscope; TGA: thermogravimetric analysis; XRD: X-ray diffraction.

## Competing interests

The authors declare that they have no competing interests.

## Authors' contributions

YZ carried out experiments, analysed and discussed data and wrote the paper; JL carried out experiments; CW carried out experiments, LG conceived, designed and carried out experiments, analysed and discussed data and wrote the paper.

## Supplementary Material

Additional file 1 **Supporting information**. Experimental details, the proof of π-π interaction between ligand and CNMs, the linkage of NPs and CNMs, electrochemical measurements and NPs on MWNTs. Click here for file

## References

[B1] Eder D Carbon Nanotube-Inorganic Hybrids Chem Rev 2010 110 1348 and the reference there in. 10.1021/cr800433k 20108978

[B2] Chu H Wei L Cui R Wang J Li Y Carbon nanotubes combined with inorganic nanomaterials: Preparations and applications Coord Chem Rev 2010 254 1117 and the reference there in. 10.1016/j.ccr.2010.02.009

[B3] Banerjee S Wong SS Synthesis and Characterization of Carbon Nanotube-Nanocrystal Heterostructures Nano Lett 2002 2 195 10.1021/nl015651n

[B4] Woan K Pyrgiotakis G Sigmund W Photocatalytic Carbon-Nanotube-TiO2 Composites Adv Mater 2009 21 2233 10.1002/adma.200802738

[B5] Hungria AB Juarez BH Klinke C Weller H Midgley PA 3-D characterization of CdSe nanoparticles attached to carbon nanotubes Nano Res 2008 1 89 10.1007/s12274-008-8011-x

[B6] Tang JM Jensen K Waje M Li W Larsen P Pauley K Chen Z Ramesh P Itkis P Yan Y Haddon RC High Performance Hydrogen Fuel Cells with Ultralow Pt Loading Carbon Nanotube Thin Film Catalysts J Phys Chem C 2007 111 17901 10.1021/jp071469k

[B7] Wildgoose GG Banks CE Compton RG Metal Nanoparticles and Related Materials Supported on Carbon Nanotubes: Methods and Applications Small 2006 2 182 10.1002/smll.200500324 17193018

[B8] Georgakilas V Gournis D Tzitzios V Pasquato L Guldi DM Prato M Decorating carbon nanotubes with metal or semiconductor nanoparticles J Mater Chem 2007 17 2679 10.1039/b700857k

[B9] Qu LT Dai LM Shape/Size-Controlled Syntheses of Metal Nanoparticles for Site-Selective Modification of Carbon Nanotubes J Am Chem Soc 2005 127 10806 10.1021/ja053479+ 16620126

[B10] Han WQ Zettl A Coating Single-Walled Carbon Nanotubes with Tin Oxide Nano Lett 2003 3 681 10.1021/nl034142d

[B11] Coleman KS Bailey SR Fogden S Green MLH Functionalization of Single-Walled Carbon Nanotubes via the Bingel Reaction J Am Chem Soc 2003 125 8722 10.1021/ja0355675 12862456

[B12] Chu HB Wang JY Ding L Yuan DN Zhang Y Liu J Li Y Decoration of Gold Nanoparticles on Surface-Grown Single-Walled Carbon Nanotubes for Detection of Every Nanotube by Surface-Enhanced Raman Spectroscopy J Am Chem Soc 2009 131 14310 10.1021/ja9035972 19764748

[B13] Li J Tang SB Lu L Zeng HC Preparation of Nanocomposites of Metals, Metal Oxides, and Carbon Nanotubes via Self-Assembly J Am Chem Soc 2007 129 9401 10.1021/ja071122v 17616130

[B14] Wang D Li ZC Chen LW Templated Synthesis of Single-Walled Carbon Nanotube and Metal Nanoparticle Assemblies in Solution J Am Chem Soc 2006 128 15078 10.1021/ja066617j 17117845

[B15] Eder D Windle AH Carbon-Inorganic Hybrid Materials: The Carbon-Nanotube/TiO_2 _Interface Adv Mater 2008 20 1787 10.1002/adma.200702835

[B16] Yang DQ Hennequin B Sacher E XPS Demonstration of π-π Interaction between Benzyl Mercaptan and Multiwalled Carbon Nanotubes and Their Use in the Adhesion of Pt Nanoparticles Chem Mater 2006 18 5033 10.1021/cm061256s

[B17] Wu CX Li JX Dong GF Guan LH Removal of Ferromagnetic Metals for the Large-Scale Purification of Single-Walled Carbon nanotubes J Phys Chem C 2009 113 3612 10.1021/jp810163u

[B18] Wu C Dong G Guan L Production of graphene sheets by a simple helium arc-discharge Physica E: Low-dimensional Systems and Nanostructures 2010 42 1267 10.1016/j.physe.2009.10.054

[B19] Cao CY Cui ZM Chen CQ Song WG Cai W Ceria Hollow Nanospheres Produced by a Template-Free Microwave-Assisted Hydrothermal Method for Heavy Metal Ion Removal and Catalysis J Phys Chem C 2010 114 9865 10.1021/jp101553x

[B20] Chen J Xu LN Li WY Gou XL α-Fe_2_O_3 _Nanotubes in Gas Sensor and Lithium-Ion Battery Applications Adv Mater 2005 17 582 10.1002/adma.200401101

[B21] Li JX Wu CY Guan LH Lithium Insertion/Extraction Properties of Nanocarbon Materials J Phys Chem C 2009 113 18431 10.1021/jp9061658

