# Supplementary material for: A general strategy for synthesis of metal oxide nanoparticles attached on carbon nanomaterials
Source: Nanoscale Res Lett. 2011 Jan 12;6(1):71. doi: 10.1186/1556-276X-6-71 (PMC3212219; doi:10.1186/1556-276X-6-71)
Supplement: Additional file 1 — Supporting information. Experimental details, the proof of π-π interaction between ligand and CNMs, the linkage of NPs and CNMs, electrochemical measurements and NPs on MWNTs. [file 1556-276X-6-71-S1.DOC]

Supporting Information for

**A General strategy for synthesis of metal oxide nanoparticles attached on carbon nanomaterials**

Yi Zhao, Jiaxin Li, Chuxin Wu, and Lunhui Guan*

State Key Lab of Structural Chemistry, Fujian Institute of Research on the Structure of Matter, Chinese Academy of Sciences, YangQiao West Road 155#, Fuzhou, Fujian 350002, P.R. China. Email: guanlh@fjirsm.ac.cn

**SI 1: Experimental details:**

In a typical experiment of preparing Fe_2_O_3_/SWNTs hybrids, 0.125 mmol phenylphosphonic acid and ferric nitrate nonahydrate (Fe (NO_3_)_3_) were dissolved in 10 mL deionized water and sonicated for 10 min to form a homogeneous solution. 5 mg purified SWNTs was added and the mixtures were sonicated until they dispersed uniformly. Then 200 mg urea was added. Finally the solution was transferred to a 25 mL Teﬂon-lined stainless-steel autoclave and maintained at 180 ℃ for 36 h. The final precipitates were filtered and washed several times with water and then dried at 80 ℃ overnight. The samples was characterized by HR-TEM ( JEOL-2010) and X-ray diffaction pattern(XRD, recorded on a MiniflexⅡ diffractometer equipped with Cu/Kα radiation λ = 0.15405 nm, 30 kV, 15 mA), and The thermogravimetric analysis (TGA, NETZSCH STA-449C).

**SI 2: The proof of π-π interaction between ligand and CNMs.**

For comparison, we performed a reference experiment by synthesizing the NPs without the phenylphosphonic acid. The typical TEM image of the obtained product shown below indicated relatively larger NPs unanchored on the SWNTs, indicating the key role of the ligand when attaching NPs on the CNMs.


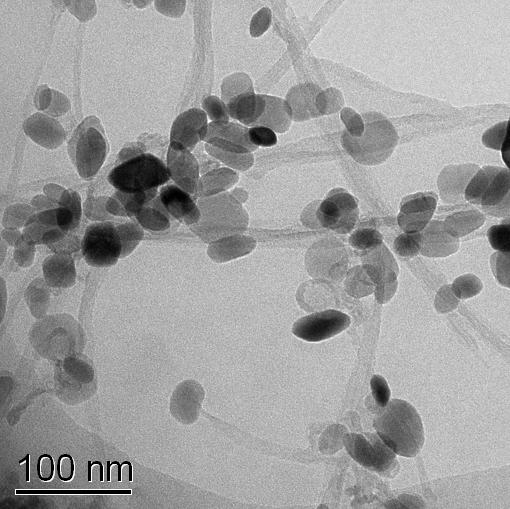


Figure S1. Larger particles sizes of the unanchored phase of Fe_2_O_3_ without phenylphosphonic acid

We also checked the intermediate product after sonication. In a typical reference experiment, 0.125 mmol ferric nitrate nonahydrate (Fe (NO_3_)_3_) were dissolved in 10 mL deionized water and sonicated for 10 min to form a homogeneous solution. 5 mg purified SWNTs was added and the mixtures were sonicated until they dispersed uniformly. Another sample was prepared by sonication of Fe(NO_3_)_3_, phenylphosphonic acid, and 5 mg SWNTs. After sonication, the products were filtered, washed with deionized water thoroughly, and dried in 80 ^o^C for 2h. The TGA measured the total metal contents with a heating rate of 10 °C /min in air. The TGA results proved that there was weak interaction between metal ions and CNMs without phenylphosphonic acid. The TGA residue (mainly iron oxide) of the products, made from SWNTs sonicated with only Fe^3+^ was nearly zero. On the contrary, that from SWNTs socinated with Fe^3+^ and phenylphosphonic acid was around 20%. The results provided direct evidence that phenylphosphonic acid acted as bridges connecting metal ions and carbon nanomaterials.


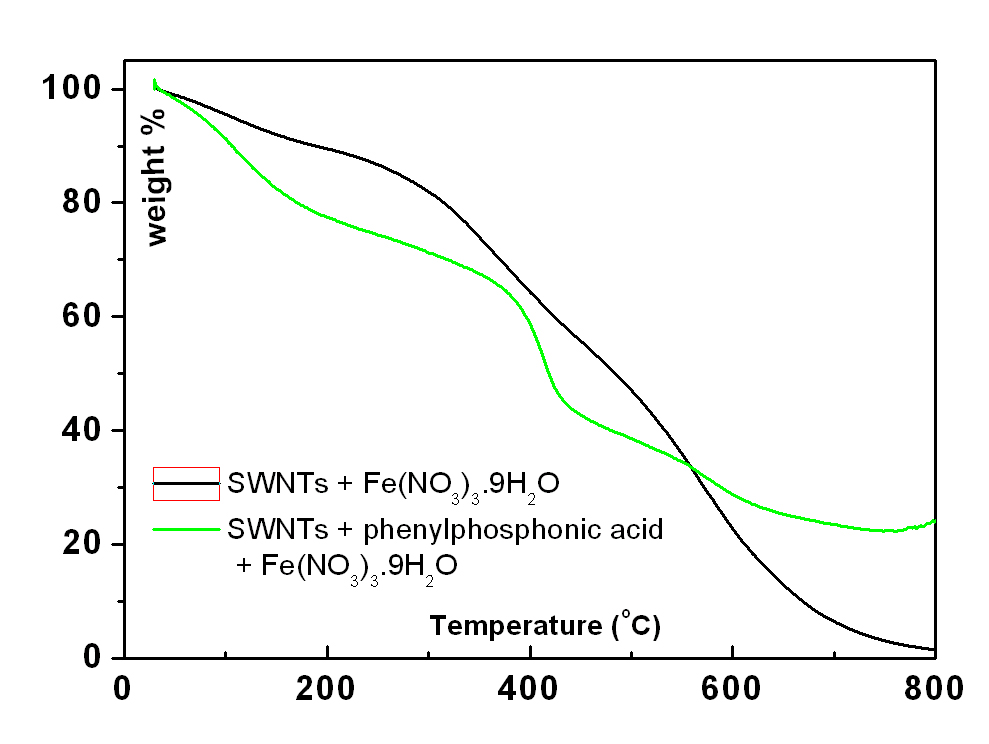


**Figure S2. TGA results of SWNTs sonicated with only Fe^3+^(black) and SWNTs sonicated with Fe^3+^ and phenylphosphonic acid (green) after washing.**

**SI 3: The linkage of NPs and CNMs**

Figure S4a shows the powder XRD pattern of four metal oxide NPs on SWNTs. For typical Fe_2_O_3_/SWNTs nanocomposites, the XRD patterns (figure S4a) of the as precipitated sample are highly intense and match well with the standard data of α-Fe_2_O_3_ (hematite, standard cards: JCPDS- 33-0664). The sharp peaks in the XRD were ascribed to the agglomerating of the NPs via oriented attachment. (J. Electrochem. Soc., 156(7) (2009) D231-D235) However, as for the rare earth metal oxide Er_2_O_3_, they formed nearly amorphous structures in such reaction conditions.

Figure S4. XRD pattern of four metal oxide NPs on SWNTs.

The loading ratio in this study was relatively high, around 80%, resulting in the agglomerating of the NPs on the CNMs. The interface between NPs and CNMs is not prominent. When we decreased the loading ratio, the uniformly dispersed NPs were appeared on the surface of CNMs. We chose SnO_2_ on SWNTs as example, the TEM image shown below indicate the clear linkage of NPs and SWNTs


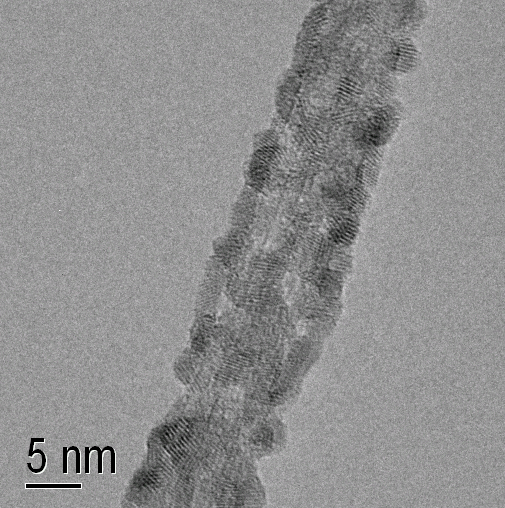


**Figure S4. HR-TEM images of SnO_2_/SWNTs with relatively lower loading ratio of SnO_2_, indicating the clear linkage of NPs and SWNTs**

**SI4: Electrochemical measurements**

The electrochemical measurements were carried out via a CR2025 coin-type test cells fabricated in a dry argon-ﬁlled glovebox. The working electrode consisted of 75 wt% active material (Fe_2_O_3_/SWNTs hybrids), 15 wt% conductivity agent (acetylene black), and 10 wt% polymer binder (polyvinylidene difluoride, PVDF). The electrolyte was 1 M LiPF6 in EC:EMC:DMC (1:1:1 in volume). Lithium sheet was used as the counter and reference electrode. The cells were discharged and charged at a constant current of 150 mA g^-1^ over a range of 0.05 v to 3.00 v.

**Figure S5. Galvanostatic discharge-charge curves of the cell with** **Fe_2_O_3_/SWNTs** **hybrids in the voltage range 0.05-3.0 V at a current of 150** **mA g^-1^.**

**SI5: NPs on MWNTs**


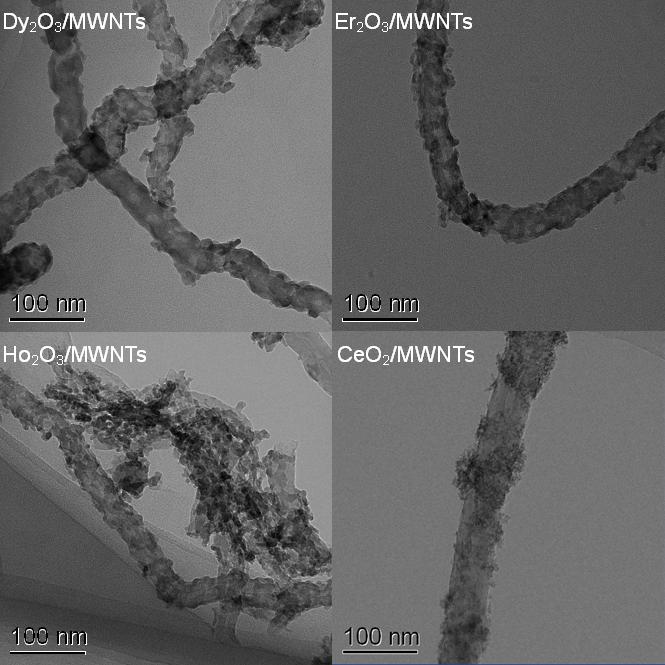
**Figure S6. TEM images of various rare earth metal oxides on MWNTs.**
